# Genetic Predisposition to a Higher Whole Body Water Mass May Increase the Risk of Atrial Fibrillation: A Mendelian Randomization Study

**DOI:** 10.3390/jcdd10020076

**Published:** 2023-02-10

**Authors:** Qi Zhu, Qiyu Chen, Ying Tian, Jing Zhang, Rui Ran, Shiyu Shu

**Affiliations:** Department of Anesthesiology, The Second Affiliated Hospital of Chongqing Medical University, Chongqing 404100, China

**Keywords:** mendelian randomization, atrial fibrillation, whole body water mass, causal association

## Abstract

Background: Observational studies have found an association between increased whole body water mass (BWM) and atrial fibrillation (AF). However, the causality has yet to be confirmed. To provide feasible protective measures on disease development, we performed Mendelian randomization (MR) design to estimate the potential causal relationship between increased BWM and AF. Methods: We implemented a two-sample MR study to assess whether increased BWM causally influences AF incidence. For exposure, 61 well-powered genetic instruments extracted from UK Biobank (*N* = 331,315) were used as the proxies of BWM. Summary genetic data of AF were obtained from FinnGen (Ncase = 22,068; Ncontrol = 116,926). Inverse-variance weighted (IVW), MR-Egger and weighted median methods were selected to infer causality, complemented with a series of sensitivity analyses. MR-Pleiotropy Residual Sum and Outlier (MR-PRESSO) and Radial MR were employed to identify outliers. Furthermore, risk factor analyses were performed to investigate the potential mechanisms between increased BWM and AF. Results: Genetic predisposition to increased BWM was demonstrated to be significantly associated with AF in the IVW model (OR = 2.23; 95% CI = 1.47–3.09; *p* = 1.60 × 10^−7^), and the result was consistent in other MR approaches. There was no heterogeneity or pleiotropy detected in sensitivity analysis. MR-PRESSO identified no outliers with potential pleiotropy after excluding outliers by Radial MR. Furthermore, our risk factor analyses supported a positive causal effect of genetic predicted increased BWM on edematous diseases. Conclusions: MR estimates showed that a higher BWM could increase the risk of AF. Pathological edema is an important intermediate link mediating this causal relationship.

## 1. Introduction

Atrial fibrillation (AF), a prevalent arrhythmia that poses a significant threat to the global public health burden, has increased the risk of cardiogenic embolic stroke [1], dementia [2] and death [3] due to irregular discharge activity. Unfortunately, with aging populations, the number of people with AF is predicted to reach 17.9 million in Europe by 2060, raising public health concerns [4]. Various risk factors have been found in previous studies: age, obesity, hypertension, diabetes, coronary heart disease, rheumatism and heart valve disease [5,6]. However, the prevention of AF is still insufficient [7]. Considering the severe threat that AF represents to human health, it is particularly imperative to uncover other modifiable risk factors contributing to the development of AF, which may facilitate effective protective strategies and reduce disabling complications of the disorder [8].

An increasing number of researches have demonstrated that owing to the convenience of measurements, anthropometric indicators (such as fat mass, fat-free mass, waist circumference, and waist-to-hip ratio) showed a tendency to be valuable predictors of AF [9,10,11]. Herein, we suspect whether other alternations of body composition could be an indicator of AF. Body water mass (BWM) can be easily obtained through bio-impedance, which correlates with several health problems (such as sleep apnea) owing to the increased amount [12]. However, there are lacking studies evaluating whether increased BWM fuels the risk of AF episodes. Recently some researches have validated that overhydration status might be responsible for the risk of AF, indicating a positive impact of increased BWM on AF [13,14]. In contrast, the decreased BWM appears to be an independent predictor for the incidence of AF in another observational study [15]. The inconsistent results of these studies might be due to the inability to avoid the influence of high correlation among different body composition characteristics in these observational studies. Additionally, owing to the interference of residual confounding and potential reverse causality, the causal relationship between increased BWM and AF remains unclear. Therefore, this study aims to decipher the causal effect of increased BWM on the risk of AF using a method free from the abovementioned drawbacks.

Mendelian randomization (MR) is a method of assessing potential causality between exposures and outcomes of interest. This approach utilizes single nucleotide polymorphisms (SNPs) as the confusion-free proxies for exposure, which effectively minimizes confounding bias and reverse causality in conventional designs [16]. Since this genetic variation is randomly allocated at fertilization before the onset of disease, such analysis mimics a randomized controlled trial (RCT) with randomly assigned SNPs in the offspring [17]. Herein, without substantial staffing resources and time-consuming subsequent tasks, MR analysis plays a crucial role in causal inference in the lack of RCTs [18]. Using the MR method, an increasing number of risk factors [19,20] for AF and the relationship between AF and other diseases [21,22] have been reported before. However, the causal effect of increased BWM on AF has not been confirmed yet. In this context, based on publicly available genome-wide association studies (GWAS) data from a large European population, we performed a two-sample MR approach to elucidate the potential causal relationship between increased BWM and AF. Furthermore, since edematous diseases such as chronic kidney disease (CKD) [23,24], type 2 diabetes (T2D) [25,26], hypertension [27,28] and heart failure [29] are well established risk factors for AF, a similar MR method was conducted in our analysis to decipher the potential mediating mechanisms on the pathway from a higher BWM to AF.

## 2. Methods

**Figure 1:**
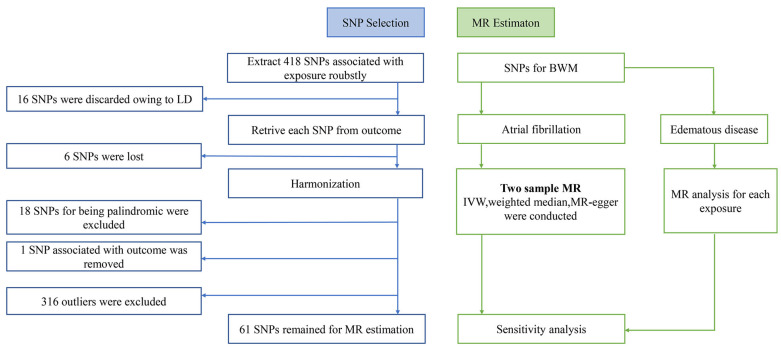
Flowchart of the present MR flame. SNP, single nucleotide polymorphism; BWM, body water mass; MR, Mendelian randomization; LD, linkage disequilibrium.

We performed a two-sample MR method to explore the potential causal relationship between increased BWM and AF. Ethical approval of our study was not required due to the absence of individual-level data. The study applied the two-sample MR package (version 0.5.6) and RadialMR package (version 1.0) of the R program (version 4.2.1) to conduct all statistical analyses. All details of datasets employed in our study are displayed in Table 1. The current MR design is shown in Figure 1.

### 2.1. Data Sources

The UK Biobank (UKB) is a large-scale and detailed prospective cohort study that explores in-depth genetic and health information in European populations to promote human healthcare and provide new insights into the prevention and treatment strategies of various chronic diseases (https://www.ukbiobank.ac.uk/, accessed on 1 October 2022). Between 2006 and 2010, over 500,000 participants aged 40–69 years were recruited to complete a range of baseline measurements [30]. The genome-wide genotyping was conducted on 488,377 participants with the UK Biobank Lung Exome Variant Evaluation (UK BiLEVE) and UK Biobank Axiom arrays. Approximately 90 million variants were imputed with the IMPUTE4 program by using the Haplotype Reference Consortium (HRC) and the merged UK10K and 1000 Genomes phase 3 reference panels [31]. BWM-related GWAS data were obtained from Neale Lab (http://www.nealelab.is/, accessed on 1 October 2022). Neale Lab implemented a GWAS analysis among thousands of human characteristics in 331,315 unrelated European populations using the data from UKB. The BWM of participants was assessed by impedance technique (measured in kg). Data were accurate to 0.1 kg. Summary-level GWAS data for AF were gained from the FinnGen biobank with up to 138,994 participants (22,068 cases with AF and 116,926 control samples without AF). According to FinnGen (https://risteys.finngen.fi, accessed on 1 October 2022), the definition of AF is “A disorder characterized by an electrocardiographic finding of a supraventricular arrhythmia characterized by the replacement of consistent *p* waves by rapid oscillations or fibrillatory waves that vary in size, shape and timing and are accompanied by an irregular ventricular response.” This disease was determined by reviewing the medical documentation based on ICD-10 criteria. Detailed information for outcome data was provided in Supplementary Table S1.

### 2.2. Selection of Genetic Instruments

**Figure 2:**
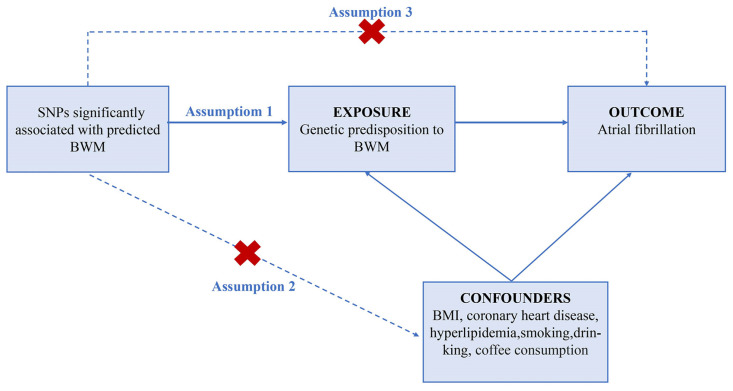
Three imperative assumptions of Mendelian randomization approach. SNP, single-nucleotide polymorphism; BWM, body water mass; BMI, body mass index.

A rigorous filtering procedure was carried out to control the quality of our analysis strictly. Only individuals of European descent were enrolled in the study to minimize potential confounding bias associated with descent. Ideally, well-powered instrumental variables (IVs) should fulfill three assumptions (Figure 2): (i) IVs are strongly related to exposure of interest (*p* < 5 × 10^–8^); (ii) IVs are independent of possible confounders; (iii) IVs should not be related to the outcome through any alternative pathway other than via a hypothesized one [32]. In accordance with the core assumptions of the MR study, we conducted a collection of filtering steps as following (Figure 1): (a) We selected valid SNPs closely associated with BWM as IVs from a summary-level GWAS dataset with a stringent threshold (*p* < 5 × 10^–8^, IV Assumption i, Figure 2). (b) To guarantee the IVs chosen for BWM are independent of each other, we set strict requirements (LD threshold of *r*^2^ < 0.001) to minimize the effect of linkage disequilibrium (LD). (c) We extracted the SNPs from the dataset of outcomes (AF). If IVs for BWM were not available in the outcome data, we then searched online (https://snipa.helmholtz-muenchen.de/snipa3/index.php, accessed on 2 October 2022) for proxy SNPs (LD threshold of *r*^2^ > 0.80). For those absent in the outcome without suitable proxies, we discarded them. (d) We excluded the palindromic and incompatible SNPs after harmonization, a process that enables the SNPs-exposure and SNPs-outcome to correspond to the same allele. In this step, we also removed SNPs related to the outcome dataset (IV Assumption iii, Figure 2). (e) To ensure the reliability of our study, Radial regression of MR (Radial MR) [33] was then performed to identify and exclude outliers with possible pleiotropy as an alternative approach to MR-pleiotropy residual sum and outlier (MR-PRESSO). *F*-statistics were performed to test the strength of chosen SNPs. Typically, *F* > 10 may indicate sufficient strength in causal Inference [34]. The flowchart of SNPs filtering is shown in Figure 2, and Supplementary Table S2 displays the final list of complex traits of selected SNPs.

### 2.3. MR Estimates

To demonstrate the causal relationship between increased BWM and AF, we conducted three primary methods in a two-sample MR analysis, namely inverse variance weighted method (IVW), weight median, and MR-Egger regression. Different methods were implemented to settle the effect of variant heterogeneity and pleiotropy due to their different fundamental assumptions. We used the IVW model as the primary estimate, a traditional approach to combine the Wald ratio of multiple SNPs and obtain a pooled causal effect by conducting a meta-analysis. The weighted median estimator provides a robust result, requiring that at least half of the SNPs used in the analysis are valid [35]. Notably, MR-Egger regression has less statistical power than IVW but provides a broader confidence interval since this method allows all IVs for horizontal pleiotropic effect [36]. In addition, we also applied other MR estimates, including simple mode and weighted mode, as complementary tools to explore the causality. The consistent results of estimates among all MR models provide credible evidence for our analysis. *p* < 0.05 was set as significance. If there are any inconsistent results among different MR models, we then tighten the *p* value threshold [37]. The statistical power of our MR estimates was calculated with a significance value of 0.05 based on Brion et al. [38] (https://shiny.cnsgenomics.com/mRnd/, accessed on 2 October 2022). Generally, adequate power of 80% or more was suggested.

### 2.4. Sensitivity Analysis

Sensitivity analysis has been a crucial device for detecting potential heterogeneity and pleiotropy in MR studies. When IVs associated with exposure (BWM) act directly on the outcome (AF) via various pathways other than the one of interest, this indicates the presence of horizontal pleiotropy. Herein, the MR-Egger intercept test (*p* > 0.05 was considered to be absent of pleiotropy), MR-Pleiotropy Residual Sum and Outlier methods (MR-PRESSO), Radial MR and funnel plot were applied to explore the underlying pleiotropy in the MR estimates. MR-PRESSO causes regression in the estimates of SNP-exposure on the estimates of SNP-outcome, aiming to detect outlier SNPs and yield calibrated causality [39]. Cochran Q-test was also conducted to appraise the heterogeneity of our MR results. Specifically, there was a presence of underlying heterogeneity in our MR analysis when *p* < 0.05 in the Cochran Q-test [40]. In addition, we used the leave-one-out (LOO) analysis to evaluate whether any single SNP determined causality by removing SNPs by turns and recomputing the result.

To check whether the core assumption of MR analysis was violated by residual confounds (IV Assumption ii, Figure 2), we also screened PhenoScanner for SNPs related to any-well accepted risk factors of AF (www.phenoscanner.medschl.cam.ac.uk, accessed on 3 October 2022) with the threshold of 1 × 10^−5^, including BMI, coronary artery heart disease (CHD), hyperlipidemia, smoking, alcohol use, and coffee intake. If any IVs associated with potential confounders were noted, we then discarded them manually and recomputed MR analysis to verify that the result was consistent.

### 2.5. Risk Factors

To reveal the potential mediating mechanisms genetically linking increased BWM and AF, we further performed the IVW estimate to illustrate the causal relationship between increased BWM and several common edematous diseases, including CKD, T2D, heart failure, and hypertension. Genome-wide summary data for heart failure and hypertension were obtained from the FinnGen dataset. The endpoint definition and detailed characteristics of these two disorders can be found online (https://risteys.finngen.fi, accessed on 1 October 2022). GWAS data for CKD were extracted from a meta-analysis of up to 133,413 participants performed by Pattarot et al. Disease is determined under the guidelines of the National Kidney Foundation [41]. For diabetes, we obtained genetic information for combined sexes from Angli Xue et al. for causal inference [42]. Details of GWAS data are shown in Table 1. Using BWM as exposure while the potential risk factors described above as outcomes, we implemented MR analysis to uncover the intermediate pathways between increased BWM and AF. Estimates from the IVW method were employed as the main results with a statistical significance of *p* < 0.05.

## 3. Results

**Figure 3:**
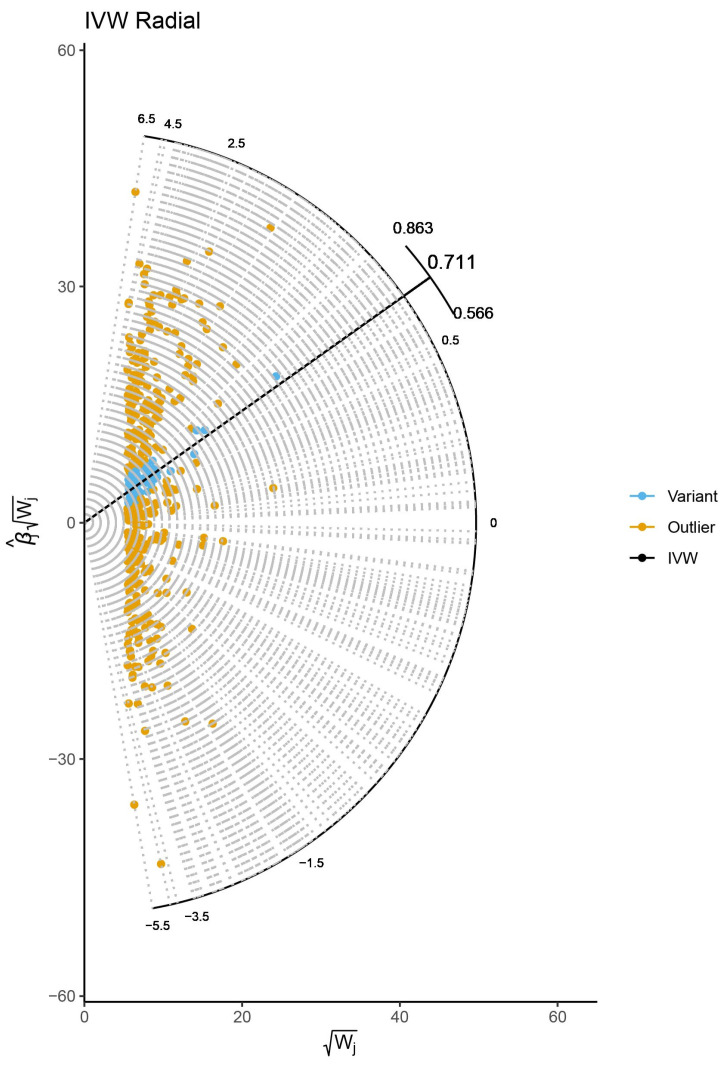
Outliers identified by Radial Mendelian randomization method. IVW, inverse variance weighted.

In the present study, we selected 61 valid SNPs as genetic instruments for predicted BWM after a range of vigorous filtering procedures (Supplementary Table S2). There were 6 SNPs lost in the process of analysis, but no appropriate proxy was noted. Radical MR estimator identified 316 outliers (Figure 3), and we removed them manually. The F-statistics for all chosen SNPs ranged from 12 to 91, indicating no weak IVs in this MR analysis.

### 3.1. Estimation of Causal Effect of BWM on AF

**Figure 4:**
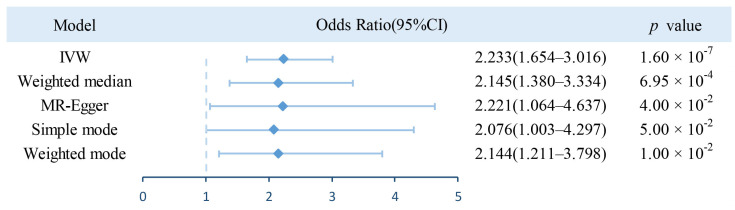
Risk of whole body water mass for genetically predicted atrial fibrillation. IVW, inverse variance weighted.

The figure displays the results of the two-sample MR analysis for a credible relationship between increased BWM and AF episodes (Figure 4). The IVW model provided robust evidence that a higher BWM can significantly increase the risk of AF (OR = 2.233; 95% CI = 1.654–3.016; *p* = 1.60 × 10^−7^). Likewise, the consistent estimates were demonstrated using MR-Egger regression (OR = 2.221; 95% CI = 1.064–4.637; *p* = 0.04) and weighted median method (OR = 2.145; 95% CI = 1.380–3.334; *p* = 6.95 × 10^−4^). Furthermore, a positive causality between genetic liability for increased BWM and AF was also detected using other approaches (*p* < 0.05 in both Simple mode and Weighted mode methods), strengthening the reliability of our instrumental-variable analysis.

To eliminate the potential confounding effect of risk factors on causal inference, we performed SNPs search in Phenoscanner. Specifically, 15 SNPs related to confounders were detected. 13 SNPs (rs111640872, rs1286138, rs2281175, rs2815753, rs545608, rs57636386, rs66922415, rs7298201, rs73052033, rs836519, rs9826759, rs9861443 and rs9951619) genetically predicted BMI; 2 SNPs (rs28391281 and rs28929474) were associated with CHD. Similar results were obtained from repeated MR analysis (OR = 2.134; 95% CI = 1.473–3.090; *p* = 6.03 × 10^−5^) after excluding these SNPs, and there was no evidence to show any existence of heterogeneity and pleiotropy. This suggested no violations from residual confounds in our analysis and the essential assumptions of MR study were met.

We implemented a range of sensitivity analyses to validate the robustness of MR analysis, including MR-Egger intercept test, MR-PRESSO, funnel plot and LOO analysis. Cochran’s Q-test did not identify any heterogeneity in IVW method (Q = 19.64; *p* = 0.99). The intercept term obtained from MR-Egger intercept test showed no evidence of possible horizontal pleiotropy (intercept = 9.09 × 10^−5^; *p* = 0.99). MR-PRESSO and Radial MR also found no valid proof of any outlier SNPs with potential pleiotropic effects. A Scatter plot of the current analysis can be found in Supplementary Figure S1. The Funnel plot (Supplementary Figure S2) was symmetrical, suggesting that no estimate was violated. Moreover, the leave-one-out analysis (Supplementary Figure S3) showed a steady estimate when throwing a single SNP one by one, indicating that the pooled IVW result was not driven by one single SNP. Power analysis revealed that our study provided sufficient power (100%) (Supplementary Table S3) to infer the impact of increased BWM on AF in the context of massive sample size (138,994) and the threshold of 0.05.

### 3.2. Risk Factor Analysis

To further explore the potential intermediating factors connecting increased BWM to AF, we conducted similar MR analyses of edematous conditions that a higher BWM may influence. Table 2 shows the results of the risk factor analysis for the association between increased BWM and edematous diseases. IVW model provided credible evidence of a causal association between genetically determined increased BWM and CKD (OR = 1.432; 95% CI = 1.231–1.667; *p* = 3.48 × 10^−6^), T2D (OR = 1.339; 95% CI = 1.166–1.537; *p* = 3.40 × 10^−5^), heart failure (OR = 1.555; 95% CI = 1.371–1.763; *p* = 5.95 × 10^−12^) and hypertension (OR = 1.119; 95% CI = 1.002–1.249; *p* = 0.046). MR-Egger intercept analysis demonstrated no presence of potential pleiotropy for those disorders, complemented with a sufficient statistical power of all results (Supplementary Table S3). Overall, based on the above analyses, our study illustrated edematous diseases such as CKD, T2D, heart failure, and hypertension might be the pivotal mediators of the causal effect of increased BWM and a higher risk of AF.

## 4. Discussion

Using large-scale GWAS data from UKB and FinnGen biobank, we applied a two-sample MR analysis to comprehensively investigate whether genetic predisposition toward BWM increases the incidence of AF. We found credible evidence to verify that the genetic liability for a higher BWM might be responsible for AF episodes. Specifically, per SD (Std.dev = 8 kg) increase in BWM was associated with a 2.233-fold higher risk of AF according to the MR method. Furthermore, to identify the potential mediating mechanisms on the pathway from increased BWM to AF, we demonstrated that genetically increased BWM was associated with several common edematous disorders, including CKD, T2D, heart failure, and hypertension according to our risk factor analyses. There is still a higher prevalence of AF in both developed and developing countries [3], and our study brings new elements to the prevention and management of AF to decrease the worldwide burden of AF and its sequelae effectively.

Atrial rhythm disturbances are a common phenomenon across the world [43], with AF being the most common type of arrhythmia. With the incidence of AF increasing over the years, it is crucial to explore the various risk factors of AF. Previous studies have demonstrated the significant contribution of cytoskeletal protein variants and calcium ions in the development of AF from a molecular biological perspective [44,45,46]. And in the present research, we focus on the role of a higher BWM in the onset of AF. However, the contrary results have been observed in clinical studies exploring the relationship between BWM and AF. In 5 hospitals in Finland, Kaartinen et al. performed a prospective study of 69 individuals in the condition of end-stage renal disease. This research provided compelling evidence that fluid overload played a critical role in AF with insertable cardiac to identify AF and body composition monitor for overhydration status detection [13]. Recently, Anaszewicz et al. implemented a study of 120 patients hospitalized for AF and 240 patients clinically diagnosed with other cardiovascular disorders. A significant association between increased BWM and increased risk of AF was elucidated by applying anthropometric examination [14]. A higher BWM may lead to maladaptive alterations in cardiac structure, such as left ventricular hypertrophy and fibrosis, facilitating the development of AF [47]. Paradoxically, according to an observational study performed by Anna et al., hospitalized heart failure patients in a dehydrated state tended to be more prone to AF [15]. The contradictory findings may be explained by the inherent faults of traditional observational studies, such as confounding factors and reverse causality in causality inferences. Moreover, there is the possibility of reverse causality even in prospective studies. Compared to the comparatively impractical large-scale prospective clinical trials that require long-term observation, our MR study revealed a positive causal relationship between increased BWM and AF in a time-conserving and low-cost manner, which was consistent with the conclusions derived from some previous studies.

Our results extend the literature on the issue of increased BWM in AF events in a new manner. The main finding of our research is that the relationship between a higher BWM and AF reported in previous observational studies is essentially causal. In the MR frame, the causality was validated by the consistent magnitude and direction estimates from five MR models (IVW, Weighted median, MR-Egger, Simple mode, and Weighted mode method), complemented with a series of sensitivity analyses as validation of primary MR results. Specifically, an increase of each SD in BWM predicted a 2.233-fold increase in the risk of AF based on the two-sample MR analysis, suggesting that we can achieve early prevention and intervention of disease by monitoring BWM. In addition, even excluding SNPs strongly correlated with confounding factors, the causal relationship remains, illustrating that increased BWM dose acts as an independent predictor of AF. Moreover, further risk factors analysis showed a robust association between genetic liability for a higher BWM and various diseases. This likewise provides new insights into the early identification of patients at higher risk.

The present analysis provided convincing evidence for a causal effect of increased BWM on AF, and various intermediary factors might mediate this relationship. The multiple potential mechanisms might be attributed to the followings (Supplementary Figure S4). First, an increasing trend of BWM may be accompanied by chronic volume overload (CVO), leading to atrial dilatation and myocardial fibrosis [48]. And it has been reported that reentrant and focal activation, commonly considered to be triggered by microreentry and unusual automaticity in CVO-induced atrial dilatation, should be responsible for the incidence of AF. The secondary slowing conduction also acts as a key player in facilitating reentry during AF [48]. Additionally, based on the results of risk factor analysis, our study deciphered that genetic liability for increased BWM was fueled by edematous diseases (CKD, T2D, heart failure, hypertension). Hence, we assume that patients with a higher BWM are more likely to develop edematous diseases, increasing their vulnerability to AF. A recent study showed that dysfunction of pericytes or endothelial cells arising from tissue edema might be a potential trigger for the onset of AF in patients exposed to COVID-19 infection [49], which supported that pathologic edema might play an influential part in the development of AF. Furthermore, as the well-recognized risk factor for AF, obesity may have a crucial mediating impact on the causal relationship between increased BWM and AF [50]. Since obese individuals generally have a higher BWM than those with average weight [51], increased BWM tends to be tied to obesity condition. The growth in body weight is paralleled by tissue fibrosis, infiltration of fat cells into the adjacent myocardium, shortened action potential interval, and epicardial fat-associated inflammatory hallmarks [52] contributing to AF susceptibility among people. Additionally, AF episodes might be fueled by the deleterious effects on profibrillatory hemodynamics, musculoskeletal and adipose tissue caused by overweight [53]. Moreover, it is noteworthy that obesity also fosters the development of diseases (such as diabetes and hypertension) and enlarges the size of the atria, facilitating AF episodes [52].

Given the growing prevalence of AF, it is imperative to recognize the modifiable risk factors linked to AF, which may help us implement targeted interventions to decrease the burden of the disease. As BWM can be obtained conveniently from impedance, the utilization of this modifiable risk factor as a potential independent indicator of AF may be efficient guidance to disease screening and therapy. According to the results of our MR analysis, there is a causal relationship between genetic predisposition toward increased BWM and AF, supported by further sensitivity analyses and power statistics. Our research presents a new paradigm for the screening, targeted therapy strategies and sequelae prevention of AF. Specifically, from the public health perspective, more concern should be given to patients with a higher BWM, and to the fact that monitoring and management of fluid overload in this population may be a vital strategy for AF prevention. Recently a meta-analysis showed that diuretics significantly decreased the incidence of new and recurrent AF [54], which is consistent with our findings. According to Solun et al., diuretics were beneficial in preventing cardiovascular complications (such as AF and stroke) in patients with hypertension [55]. Furthermore, the risk of AF in individuals who reported a history of heart failure was effectively reduced by using diuretics [54]. All these studies indicated that effective fluid management was essential to prevent the development of AF. Further studies are required to evaluate the efficiency of different diuretics response to AF and its long-term sequelae such as stroke. During the therapeutic phase of the disease, the treatment modalities of AF should also consider a new pillar targeting fluid management, which may be an effective element of the multi-modality therapeutic intervention of AF. Moreover, a study has shown that increased physical activity positively reduces the incidence of AF, while obesity and edema status are generally accompanied by sedentary activity [52]. Consequently, lifestyle interventions, including losing weight and increased physical exercise [8] should be taken into account while setting a new management paradigm of AF for the patients with obesity or edema.

To the best of our knowledge, it is the first analysis to systematically appraise the casual association between genetically determined increased BWM and AF by using the MR method. Our research has several strengths. First, the analytical method employed in this study allows for mimicking RCTs in an observational environment. Secondly, using reliable genetic instruments (*F* statistic > 10) as the proxy for BWM in a robust MR framework (power statistics = 100%), our analysis minimized the bias of reverse causality and underlying confounders commonly seen in observational studies. Finally, we deployed a range of sensitivity analyses to detect the robustness of the results and minimize the interference of horizontal pleiotropy. The present analysis revealed a significant causality between genetically increased BWM and AF, providing a new insight into the refinement of intervention strategies.

## 5. Limitation

Notably, there are still some things that could be improved in our analysis. First, our MR analysis comprised only European participants to maintain a consistent genetic background. Therefore, the outcomes may not present as applicable to other races. Second, although we excluded a series of common confounders in our sensitivity analysis, some unidentified potential confounders that were not removed might still exist, limiting the capability of avoiding the confounding bias of each SNP. Third, summary-level data were employed in the current study for causal inference. Hence, we could not exclude the probability that the causal effect of increased BWM on AF might be non-linear. Finally, our research on the potential mechanisms mediating this causal effect is only an incomplete exploration. Therefore, more researches are warranted to shed light on the detailed biological mechanisms underlying this causal relationship in future research.

## 6. Conclusions

In summary, our MR study demonstrates that genetic liability for a higher BWM may increase the risk of AF, in which pathological edema is a crucial potential mediator of this causal relationship. Further studies are needed to explore the possible non-linear association, evaluate the efficiency of different diuretics for a more optimized treatment option and illustrate various potential mechanisms.

## Figures and Tables

**Table 1:** Details of the GWASs included in the Mendelian randomization. M, male; F, female.

Consortium /Pubmed ID	Phenotype	Participants		Sex	Ancestry
		Number of Cases	Number of Controls		
Neale lab	Whole body water mass	331,315		M/F	European
FinnGen	Atrial fibrillation and flutter	22,068	116,926	M/F	European
26831199	Chronic kidney disease	12,385	104,780	M/F	European
30054458	Type 2 diabetes	62,892	596,424	M/F	European
FinnGen	Heart failure, strict	13,087	195,091	M/F	European
FinnGen	Hypertension	55,917	162,837	M/F	European

**Table 2:** Mendelian randomization estimates of the associations from whole body water mass on common edematous diseases. IVW, inverse variance weighted.

Outcomes	IVW		MR-Egger Method	
	Causa Effect (95% CI)	*p*	Intercept	*p*
Chronic kidney disease	1.432 (1.231–1.667)	3.48 × 10^−6^	−0.001	7.3 × 10^−1^
Type 2 diabetes	1.339 (1.166–1.537)	3.40 × 10^−5^	0.004	1.4 × 10^−1^
Heart failure	1.555 (1.371–1.763)	5.95 × 10^−12^	0.00096	6.9 × 10^−1^
Hypertension	1.119 (1.002–1.249)	4.60 × 10^−2^	0.001	5.5 × 10^−1^

## Data Availability

Data available in a publicly accessible repository that does not issue DOIs. Publicly available datasets were analyzed in this study. This data can be found here: (https://www.mrbase.org/, accessed on 1 October 2022).

## Supplementary Materials

The following supporting information can be downloaded at: https://www.mdpi.com/article/10.3390/jcdd10020076/s1, Figure S1: Scatter plot of SNPs associated with whole body water mass and the risk of atrial fibrillation; Figure S2: Funnel plot of SNPs associated with body water mass and the risk of atrial fibrillation; Figure S3: Leave-One-Out of SNPs associated with whole body water mass and the risk of atrial fibrillation; Figure S4: Potential mediating mechanisms on the pathway from whole body water mass to atrial fibrillation; Table S1: Key figures and longitudinal metrics of atrial fibrillation from FinnGen; Table S2: 61 SNPs were employed as instrumental variables for investigating the association between whole body water mass with atrial fibrillation after outlier exclusion; Table S3: Statistical power on all MR analysis (two-sided α = 0.05).
