# Supplementary material for: Genetic Predisposition to a Higher Whole Body Water Mass May Increase the Risk of Atrial Fibrillation: A Mendelian Randomization Study
Source: J Cardiovasc Dev Dis. 2023 Feb 10;10(2):76. doi: 10.3390/jcdd10020076 (PMC9966889; doi:10.3390/jcdd10020076)
Supplement: Supplementary file 1 [file jcdd-10-00076-s001.zip › jcdd-2172038-supplementary.pdf]

Supplementary Material

Genetic predisposition to whole body water mass may increase the risk of atrial fibrillation: A Mendelian Randomization study

Supplementary Figure S1. Scatter plot of SNPs associated with whole body water mass and the risk of atrial fibrillation.

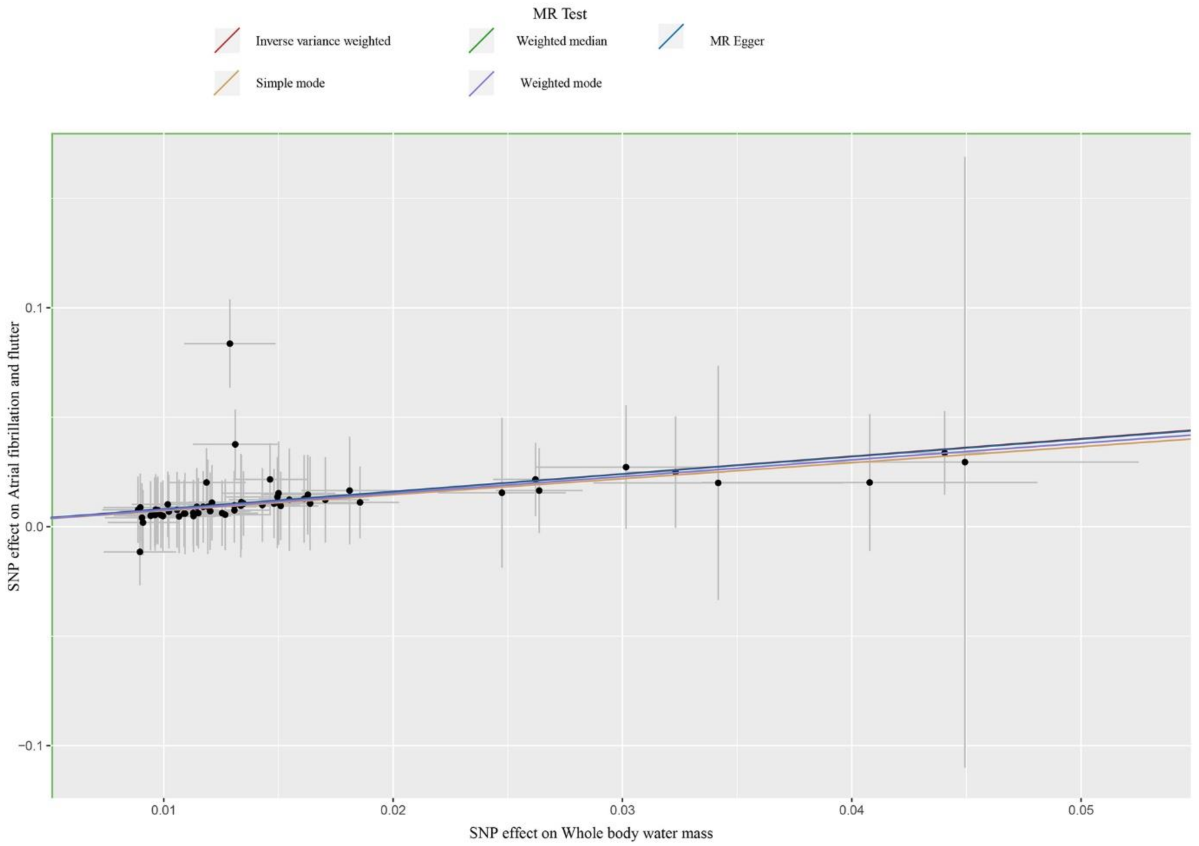

Supplementary Figure S2. Funnel plot of SNPs associated with body water mass and the risk of atrial fibrillation.

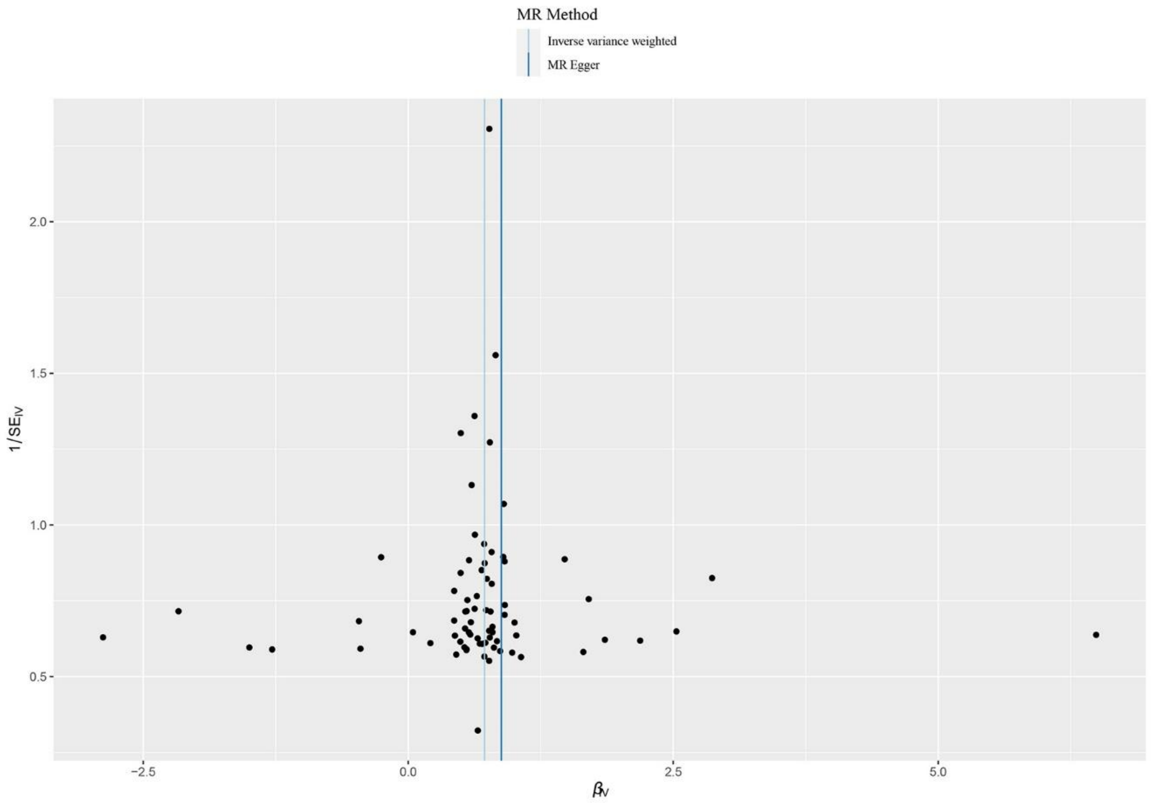
MR Method
Inverse variance weighted
MR Egger
2.0
1.5
1.0
0.5
1/SE_IV
-2.5
0.0
2.5
5.0
β_IV

Supplementary Figure S3. Leave-One-Out of SNPs associated with whole body water mass and the risk of atrial fibrillation. OR, odds ratio; CI, confidence interval.

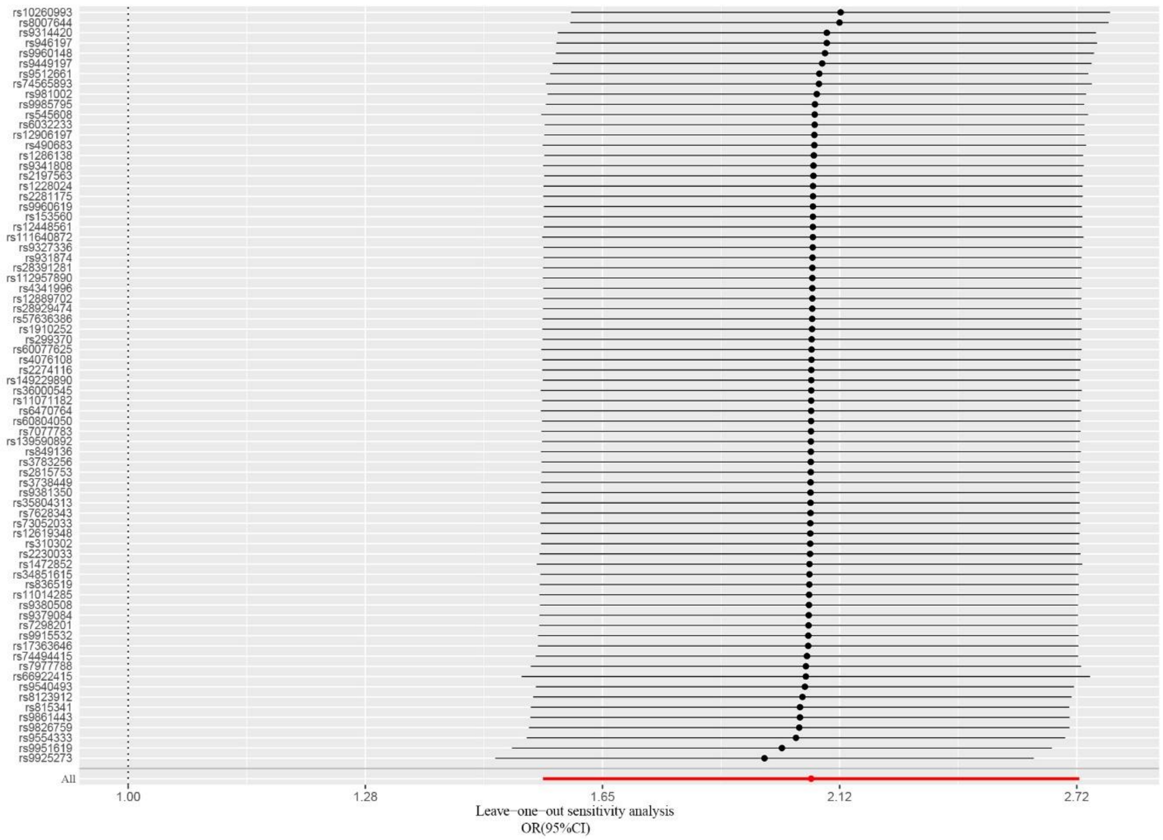

Supplementary Figure S4. Potential mediating mechanisms on the pathway from whole body water mass to atrial fibrillation. BWM: body water mass.

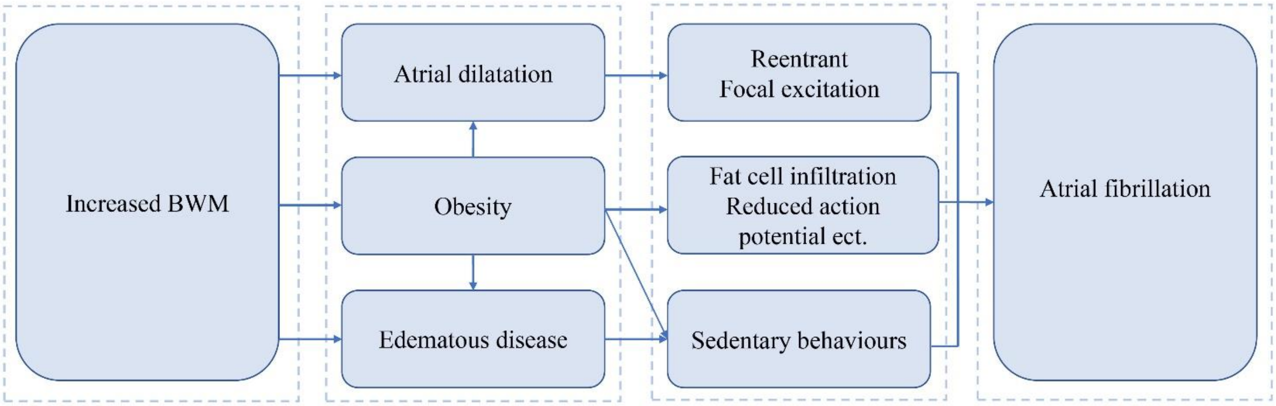

Supplementary Tables

Supplementary Table S1. Key figures and longitudinal metrics of atrial fibrillation from FinnGen.

Variables	All	Female	Male
Number of individuals	22,068	8,847	13,221
Unadjusted prevalence (%)	10.17	7.20	14.04
Mean age at first event (years)	66.70	69.42	64.89
Case fatality at 5-years (%)	9.69	9.07	10.11
Median number of events per individual	3.0	3.0	3.0
Recurrence at 6 months (%)	56.98	56.08	57.58

Supplementary Table S2. 61 SNPs were employed as instrumental variables for investigating the association between whole body water mass with atrial fibrillation after outlier exclusion. EA, effect allele; OA, other allele; EAF, effect allele frequency; SE, standard error.

No.	SNP	EA	OA	EAF	Beta	SE
1	rs11014285	A	G	0.1655	0.0181	0.0021
2	rs11071182	G	A	0.8711	0.0134	0.0023
3	rs111640872	C	G	0.3311	0.0151	0.0016
4	rs112957890	G	A	0.2658	0.0120	0.0018
5	rs1228024	A	C	0.6611	-0.0115	0.0016
6	rs12448561	C	G	0.4187	0.0100	0.0016
7	rs12619348	T	C	0.1297	-0.0134	0.0023
8	rs1286138	G	T	0.6734	0.0113	0.0016
9	rs12889702	C	A	0.3105	0.0096	0.0017
10	rs12906197	T	C	0.4248	-0.0125	0.0016
11	rs139590892	A	G	0.1574	-0.0119	0.0021
12	rs1472852	A	C	0.1564	-0.0323	0.0021
13	rs149229890	T	G	0.0112	0.0449	0.0076
14	rs153560	A	G	0.6056	0.0109	0.0016
15	rs17363646	G	A	0.1346	0.0150	0.0023
16	rs1910252	T	C	0.1641	0.0164	0.0021
17	rs2197563	A	G	0.5958	0.0107	0.0016
18	rs2230033	A	G	0.5653	-0.0135	0.0016

19	rs2274116	T	C	0.3439	-0.0096	0.0016
20	rs2281175	C	T	0.4057	0.0113	0.0016
21	rs2815753	A	G	0.5989	0.0097	0.0016
22	rs28391281	C	T	0.4598	-0.0096	0.0015
23	rs28929474	T	C	0.0204	0.0342	0.0054
24	rs310302	A	G	0.4175	-0.0089	0.0016
25	rs34851615	A	G	0.3537	-0.0090	0.0016
26	rs35804313	T	C	0.2153	-0.0114	0.0019
27	rs36000545	G	A	0.3929	-0.0148	0.0016
28	rs3738449	A	G	0.3313	-0.0117	0.0016
29	rs3783256	C	T	0.6480	-0.0097	0.0016
30	rs4076108	T	A	0.2457	0.0102	0.0018
31	rs4341996	C	A	0.2108	-0.0109	0.0019
32	rs490683	C	G	0.2908	0.0186	0.0017
33	rs545608	C	G	0.2081	0.0264	0.0019
34	rs57636386	C	T	0.0834	-0.0247	0.0028
35	rs60077625	A	G	0.3160	0.0143	0.0017
36	rs6032233	C	T	0.8083	0.0127	0.0020
37	rs60804050	A	G	0.2536	-0.0106	0.0018

38	rs6470764	T	C	0.2024	-0.0170	0.0019
39	rs66922415	G	A	0.2344	0.0440	0.0018
40	rs7077783	T	C	0.1439	-0.0150	0.0022
41	rs7298201	T	C	0.4976	0.0102	0.0016
42	rs73052033	C	T	0.1842	-0.0161	0.0020
43	rs74494415	T	C	0.0402	-0.0302	0.0039
44	rs74565893	T	C	0.0112	-0.0408	0.0073
45	rs7628343	C	T	0.9059	0.0155	0.0026
46	rs7977788	A	G	0.2248	0.0262	0.0018
47	rs836519	T	C	0.1960	0.0121	0.0019
48	rs849136	G	A	0.7132	-0.0131	0.0017
49	rs931874	T	C	0.3784	0.0094	0.0016
50	rs9327336	C	T	0.3451	0.0099	0.0016
51	rs9341808	A	C	0.4802	-0.0131	0.0015
52	rs9379084	A	G	0.1172	-0.0150	0.0025
53	rs9381350	A	T	0.3486	0.0096	0.0016
54	rs9826759	T	C	0.3533	0.0146	0.0016
55	rs9861443	C	A	0.7137	0.0119	0.0017
56	rs9915532	G	A	0.8290	-0.0163	0.0020

57	rs9925273	G	A	0.1838	-0.0129	0.0020
58	rs9951619	G	T	0.7694	0.0131	0.0018
59	rs9960148	T	G	0.3933	-0.0090	0.0016
60	rs9960619	T	C	0.3446	0.0090	0.0016
61	rs9985795	C	T	0.4811	-0.0091	0.0015

Supplementary Table S3. Statistical power on all MR analysis (two-sided α=0.05). R2: Proportion of variance explained for the association between the SNP or allele score and the exposure variable. IVW, inverse variance weighted method; OR, odds ratio; AF, atrial fibrillation; CKD, chronic kidney disease.

Outcomes	R^2	Proportion of cases	OR(IVW)	Power	Sample size required for 80% power
AF	0.0048	0.159	2.233	1.00	9,872
CKD	0.0237	0.106	1.432	1.00	20,150
Type 2 diabetes	0.0122	0.095	1.339	1.00	68,733
Heart failure	0.0175	0.063	1.555	1.00	25,973
Hypertension	0.0164	0.256	1.119	0.86	187,940
